# High-performance Mach–Zehnder modulator using tailored plasma dispersion effects in an ITO/graphene-based waveguide

**DOI:** 10.1038/s41598-022-17125-y

**Published:** 2022-07-26

**Authors:** Sohrab Mohammadi-Pouyan, Shahram Bahadori-Haghighi, Mohsen Heidari, Derek Abbott

**Affiliations:** 1 School of Electrical and Computer Engineering, Shiraz University, Shiraz, 71348-51154 Iran; 2 School of Electrical and Computer Engineering, Tarbiat Modares University, Tehran, 14115-116 Iran; 3 School of Electrical and Electronic Engineering, The University of Adelaide, Adelaide, SA 5005 Australia

**Keywords:** Optics and photonics, Optical materials and structures

## Abstract

A high-performance electro-optic Mach–Zehnder modulator (MZM) with outstanding characteristics is proposed. The MZM is in a push-pull configuration that is constructed using an ITO/graphene-based silicon waveguide. A novel idea for engineering of the plasma dispersion effect in an ITO/graphene-based waveguide is proposed so that the modulation characteristics of the MZM are highly improved. Plasma dispersion effects of ITO and graphene layers are tailored in such a way that a large difference between real parts of guided mode effective index of the two arms is achieved while their corresponding imaginary parts are equal. As a result, a very low ${{\mathrm {V}}}_{\pi }L_{\pi }$ of $10 \, \hbox {V} \upmu \hbox {m}$ is achieved. To the best of our knowledge, this is one of the lowest ${{\mathrm {V}}}_{\pi }L_{\pi }$ reported for an electro-optic modulator. In addition, the proposed modulator exhibits a very high extinction ratio of more than 30 dB, low insertion loss of 2.8 dB and energy consumption of as low as 10 fJ/bit, which are all promising for optical communication and processing systems.

## Introduction

Optical modulators are the key components of photonic communication networks and processing systems. The input information of the modulators can be transferred to the amplitude, phase or polarization of the optical carrier through electro-optic^1–5^, electro-absorption (EA)^6–8^, magneto-optic^9–11^ or thermo-optic^12–15^ effects. Optical modulators have already been investigated by many research groups^16–20^. The demand for high speed, low power consumption, high modulation depth and compact optical modulators still continues to support rapidly growing data in integrated optical systems.

Silicon photonics is a promising technology that has received tremendous interest for realizing efficient integrated optical devices such as modulators^21, 22^. However, pure silicon optical modulators are mainly based on the weak plasma dispersion effect, which hinders the realization of compact and integrated interconnections^7^. As a result, new materials and structures have already been explored to mitigate the drawbacks of silicon optical modulators. One of the outstanding materials that could revolutionize silicon photonics is graphene.

Graphene is a two-dimensional (2D) sheet of carbon atoms arranged in a honeycomb lattice that exhibits unique electrical, mechanical, thermal and optical properties^23^. The superb optical characteristics of graphene have made it an amazing choice for various applications such as photodetectors^24, 25^, logic gates^26, 27^, polarizers^15, 28^, plasmonic tweezers^29^, isolators^30^, mode-locked lasers^31, 32^, switches and modulators^13, 33–37^. The complementary metal-oxide-semiconductor (CMOS) compatibility and great tenability of graphene are of exceptional importance for optical modulators. In addition, high carrier mobility of more than $200,000 \, \hbox {cm}^2$/V s is reported in the literature and is needed to realize high bandwidth. Hence, in recent years, different kinds of graphene-based optical modulators have been studied. A double-layer graphene-based EA modulator was proposed in 2012 where a modulation depth of $0.16 \, \hbox {dB}/\upmu \hbox {m}$ at a modulating voltage of 5 V was obtained^19^. The modulator was performed by applying an external voltage across the graphene layers and changing the Fermi levels so that the absorption mechanisms were tuned. However, the modulation bandwidth was limited to 1 GHz. In 2016, a broadband graphene-based optical modulator was proposed where a modulation depth of 2 dB and insertion loss of 0.9 dB at a very high bandwidth of 35 GHz were reported^38^. However, the high bandwidth was achieved at the cost of high drive voltage of 25 V. It should be emphasized that most of the previously proposed graphene modulators have been based on the EA effects while the electro-refractive effect in graphene may also lead to high-performance modulators and phase shifters^39, 40^.

Indium tin oxide (ITO) as a transparent conductive oxide (TCO) is another material for next-generation optical modulators. The carrier concentration in ITO can be several orders of magnitude higher than silicon which leads to significant index modulation. Furthermore, the permittivity of ITO can be effectively tuned through an external bias voltage so that its real part can cross the zero point known as epsilon-near zero (ENZ). Various EA optical modulators using the ENZ behavior of ITO have been investigated^41–43^. The ITO-based EA modulators work within the α-dominant region of ITO permittivity where the optical loss is changed by modulating voltage. In contrast, the n-dominant region of ITO permittivity is of interest for phase shifters and electro-refractive modulators. However, according to Kramers–Kronig relations^44^, the real part variations of ITO refractive index is inevitably accompanied by the variations of optical loss α. Therefore, it is important to design electro-refractive modulators with low required voltage and simultaneously low insertion loss. In recent years, different kinds of electro-refractive modulators based on ITO have been studied. In 2018, an ITO-based Mach–Zehnder modulator (MZM) was proposed where a half-wave voltage and active device length product of ${{\mathrm {V}}}_{\pi }L_{\pi }=0.52 \, \hbox {V} \hbox {mm}$ was achieved^45^. However, the switching speed was undesirably limited to 1 kHz. In 2018, another research group presented a lower ${{\mathrm {V}}}_{\pi }L_{\pi }$ of $0.1 \, \hbox {V} \hbox {mm}$ in an ITO electro-optic phase modulator by which a much higher switching speed in the order of 50 GHz was estimated^46^. However, such a ${{\mathrm {V}}}_{\pi }L_{\pi }$ is still high and it is not significant. Amin et al. proposed a MZM based on a lateral ITO configuration^47^. It was experimentally shown that the ${{\mathrm {V}}}_{\pi }L_{\pi }$ product of as low as $63 \, \hbox {V} \upmu \hbox {m}$ was attainable. In 2020, a fast ITO MZM was also demonstrated with a switching speed of 1.1 GHz and an extinction ratio (ER) of 8 dB at a low ${{\mathrm {V}}}_{\pi }L_{\pi }$ of $95 \, \hbox {V} \upmu \hbox {m}$^48^.

In this paper, a very high performance ITO/graphene-based electro-optic MZM is proposed. The modulator is made of an engineered silicon waveguide based on two graphene sheets and two ITO layers as active materials whose characteristics are modified by an external voltage. The free-carrier dispersion effects in ITO and graphene are engineered so that large real part variations of guided mode effective indices in the two arms are obtained while their imaginary parts are the same. As a result, a ${{\mathrm {V}}}_{\pi }L_{\pi }$ of as low as $10 \, \hbox {V} \upmu \hbox {m}$ is achieved that is the lowest value ever reported in the literature. According to the calculations, the proposed modulator exhibits a very high extinction ratio of more than 30 dB and a low insertion loss of less than 2.8 dB. Furthermore, the calculated energy consumption of as low as 10 fJ/bit and high 3 dB bandwidth of 6.5 GHz make our proposed modultaor promising for efficient high-speed photonic circuits.

## Results

### Modulator structure and waveguide design

**Figure 1:**
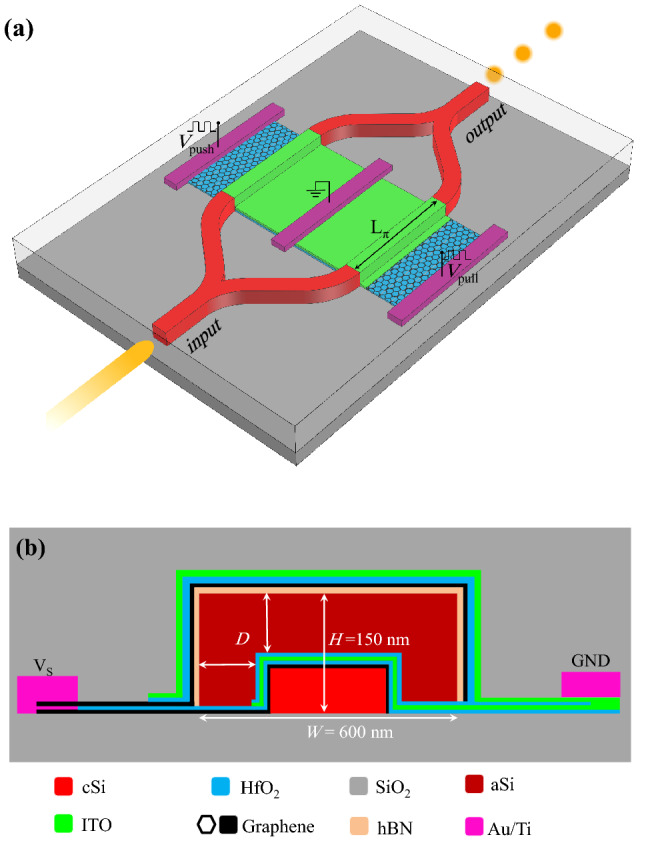
(**a**) Perspective view of the MZM in push-pull configuration. (**b**) Cross section view of the proposed ITO/graphene-based waveguide.

The perspective view of the proposed MZM is schematically shown in Fig. 1a. As it is seen, there is a phase modulating region in each arm of the MZM which is made by the proposed waveguide. The cross-section view of the waveguide is also shown in Fig. 1b, where the geometrical parameters are indicated. As it is shown, the waveguide consists of a crystalline silicon (cSi) ridge supported by a silica substrate. A stack of graphene/$\hbox {HfO}_2$/ITO/$\hbox {HfO}_2$ is grown over the cSi ridge and an amorphous silicon (aSi) layer covers the stack. A thin hexagonal boron nitride (hBN) layer is also grown on the aSi which provides a flat surface free of dangling bonds for another graphene sheet^49^. A graphene sheet is transferred to the hBN and an ITO layer is subsequently grown that are spaced by $\hbox {HfO}_2$ and the whole structure is totally covered by a silicon dioxide ($\hbox {SiO}_2$) passivation layer. In practice, the graphene film is actually transferred from a catalyst to the photonic wafer.

The total width and height of the ridge are respectively taken as $W=600 \, \hbox {nm}$ and $H=150 \, \hbox {nm}$ so that a single transverse electric (TE) mode waveguide at the operating wavelength of 1550 nm is created. The thicknesses of ITO, hBN and $\hbox {HfO}_2$ are all fixed at 5 nm. The thickness (*D*) of aSi is firstly assumed to be 100 nm but it will be optimized in the following section. It should be emphasized that in practice graphene sheets cannot be laminated on sharp corners and on 90° waveguide edges practically. However, we first assume an ideal waveguide with rectangular cross section. The practical aspects of the modulator including the waveguide with trapezoidal cross section is investigated at the end of the paper.

As it is shown in Fig. 1b, the ITO and graphene layers spaced by $\hbox {HfO}_2$ in each stack form an oxide capacitor. Two titanium-gold electrodes are located at the two sides of the waveguide with a distance of 1200 nm in order to apply the external voltage. The left and right electrodes are respectively connected to the two graphene sheets and two ITO layers.

**Figure 2:**
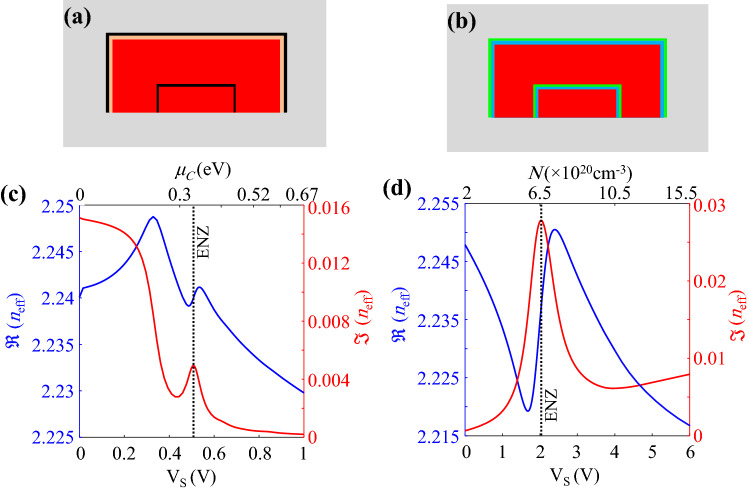
Cross section of the hypothetical waveguide (**a**) with a stack of cSi/graphene/aSi/hBN/graphene and without any ITO layers, and (**b**) with a stack of cSi/$\hbox {HfO}_2$/ITO/aSi/ $\hbox {HfO}_2$/ITO and without graphene sheets. Variations of real and imaginary parts of the effective index for TE mode of the waveguide, (**c**) in part (**a**) versus the applied voltage and graphene chemical potential, and (**d**) in part (**b**) versus the applied voltage and ITO carrier concentration.

In order to gain a better insight into the operation of the proposed waveguide, the effects of graphene sheets and ITOs on the guided mode should be investigated, separately. Hence, the stacks of the waveguide are theoretically separated to form the hypothetical waveguides shown in Fig. 2a and b. As it is shown, only the two graphene sheets and the hBN layer from the main stacks in Fig. 1b exist in Fig. 2a. In contrast, according to Fig. 2b, the structure contains the ITO and $\hbox {HfO}_2$ layers while there is no graphene sheet.

In order to calculate the guided mode effective index of the waveguide shown in Fig. 2a, the dielectric constant of graphene is obtained from Eqs. (1) and (2) (see “Methods”). In this regard, the real and imaginary parts of the TE mode effective index versus the graphene chemical potential are calculated and plotted in Fig. 2c. It should be noted that there exists a voltage axis which indicates the corresponding required voltages to attain the specified chemical potentials of graphene in the main structure of Fig. 1b. The voltages are obtained for $\hbox {HfO}_2$ thicknesses of 5 nm. Moreover, the maximum voltage of 1 V in Fig. 2c leads to the chemical potential of 0.67 eV. Although graphene chemical potentials larger than 0.6 eV are not easily attainable, there are various practical^37, 50^ and theoretical^51, 52^ works where chemical potentials of 0.7 eV to 1 eV have been reported. However, our proposed modulator can also operate with lower voltages and chemical potentials. Furthermore, assuming $\hbox {HfO}_2$ breakdown electric field of 13 MV/cm^53^, a breakdown voltage of 6.5 V is obtained for the $\hbox {HfO}_2$ layer with the thickness of 5 nm. Therefore, an applied voltage of 1 V is much lower than the breakdown voltage of $\hbox {HfO}_2$.

As it is shown in Fig. 2c, there is a weak Lorentzian oscillation around the chemical potential of 0.5 eV that is actually the ENZ point of graphene. It should be emphasized that generally, TE modes could effectively interact with the vertical graphene sheets. However, if such an interaction were dominant, a strong Lorentzian oscillation would be expected in the effective index of the TE mode around the ENZ point. Therefore, the weak oscillation shown in Fig. 2c confirms that the horizontal graphene-TE mode interaction dominates because the vertical graphene layers are located at the side walls where the electromagnetic fields are weak.

According to Fig. 2c, when the chemical potential of graphene is low, the imaginary part of the effective index is high which represents high optical loss of the guided mode due to the interband absorption of graphene sheets. It is obvious that when the chemical potential increases, the interband transitions in graphene are Pauli blocked so that the optical loss is lowered.

As a general rule, a large real part change of the effective index is of interest for an electro-refractive modulator while the imaginary parts at the two states of modulator should be nearly equal. Therefore, as it is shown in Fig. 2c, the only two points with the same values of $\Im (n_{\mathrm{eff}})$ occur near the Lorentzian oscillation around the ENZ point. However, the variations of $\Re (n_{\mathrm{eff}})$ is not considerably high to lead a very low ${{\mathrm {V}}}_{\pi } L_{\pi }$ in the modulator.

The effective index for the guided mode of the structure shown in Fig. 2b as a function of the required voltage to induce the desired carrier concentration in ITO is plotted in Fig. 2d. The induced carrier concentration in ITO is calculated from Eq. (3) (see “Methods”).

**Figure 3:**
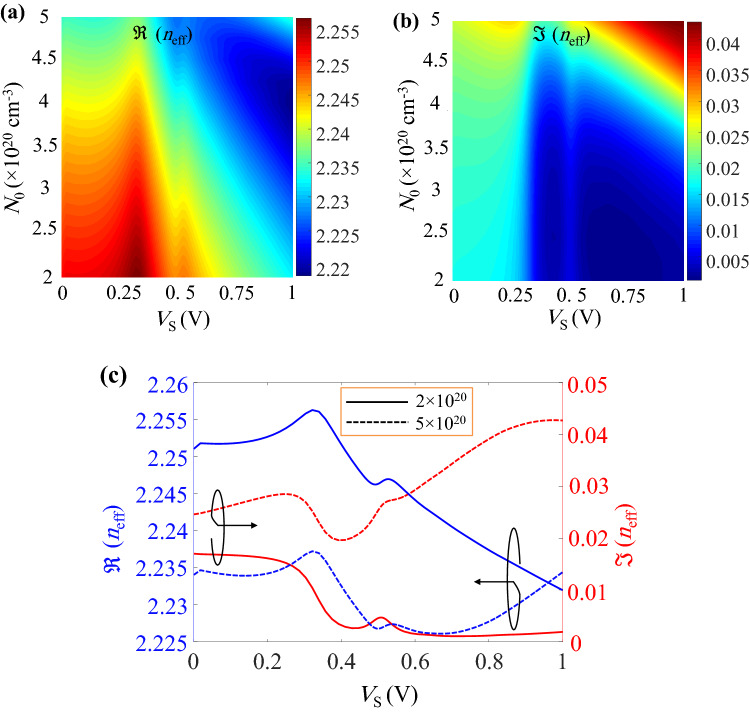
Color plots of (**a**) $\Re (n_{\mathrm{eff}})$ and (**b**) $\Im (n_{\mathrm{eff}})$ of the proposed waveguide as functions of ITO doping and the external voltage. (**c**) Variations of $\Re (n_{\mathrm{eff}})$ and $\Im (n_{\mathrm{eff}})$ at lower and upper bounds of $N_0$ (i.e. $N_0=2\times 10^{20} \, {{\mathrm {cm}}}^{-3}$ and $5\times 10^{20} \, {{\mathrm {cm}}}^{-3}$).

As it is shown in Fig. 2d, there is a strong Lorenzian oscillation around the ENZ voltage of 2 V where the ITO carrier concentration of $6.5\times 10^{20} \, {{\mathrm {cm}}}^{-3}$ is obtained. Such a Lorentzian behavior is due to the strong interaction between the TE guided mode and the 1 nm-thick accumulation layers in vertical ITOs at the side walls. It should be mentioned that the ENZ voltage of the ITO can interestingly be adjusted by the changing the initial ITO carrier concentration.

According to Fig. 2d, the two points with equal values of $\Im (n_{\mathrm{eff}})$ and large change of $\Re (n_{\mathrm{eff}})$ can be found around the ENZ point and at the external voltages of greater than 1 V. However, an undesirable energy consumption is expected as it is proportional to square of the applied voltage. Although the ITO ENZ point could be shifted toward the lower voltages by increasing its initial doping, the required voltage to achieve the best electro-refractive effect with the same imaginary parts of $n_{{{\mathrm {eff}}}}$ would remain higher than 1 V. Furthermore, the insertion loss (IL) of the waveguide increases by lowering the ENZ point that is not favorable. Therefore, according to the above discussion, neither the graphene sheets nor the ITO layers can solely result in the desired characteristics required for an efficient electro-refractive modulator. In the following discussion, it will be demonstrated that the contributions of both the ITO and graphene layers in the proposed waveguide shown in Fig. 1 result in the desired electro-refractive effect.

As it is shown in Fig. 1a, the proposed MZM is in the push-pull configuration so that two appropriate voltages of $V_{\mathrm{{push}}}$ and $V_{\mathrm{{pull}}}$ are respectively applied to the upper and lower arms of MZM. Obviously, the desired phase changes of the propagating lights are induced by the external voltages. The transmittance of the MZM can be expressed as Eq. (8) (see “Methods”). When the modulator is in the on state ($V_{\mathrm{{push}}}=V_{\mathrm{{pull}}}$) there is no phase difference between the two arms and a constructive interference occurs at the output of the MZM. At this condition, the absorption coefficients of the two arms are equal and should be low enough to ensure the low IL of the MZM. When the MZM is in the off state ($V_{\mathrm{{push}}}\ne V_{\mathrm{{pull}}}$), the applied voltages should be chosen in such a way that the largest difference between $\Re (n_{\mathrm{eff}})$ of the two arms are obtained while their absorption coefficients are the same. Obviously, the larger difference between $\Re (n_{\mathrm{eff}})$ leads to the shorter length of the modulator to achieve the required $\pi$ phase shift. Consequently, the IL and the energy consumption are also reduced.

As it is shown in Fig. 2c, $\Re (n_{\mathrm{eff}})$ and $\Im (n_{\mathrm{eff}})$ of the hypothetical waveguide with graphene sheets generally decrease by increasing the applied voltage from 0 to 1 V. On the other side, according to Fig. 2d, $\Re (n_{\mathrm{eff}})$ ($\Im (n_{\mathrm{eff}})$) of the guided mode within the assumed waveguide with ITO layers decreases (increases) for the voltage range of 0 to 1 V. Therefore, it can be concluded that by an appropriate initial doping of ITO in the proposed waveguide, it is possible to change the downward variations of $\Im (n_{\mathrm{eff}})$ (provided by the contribution of graphene sheets) to an upward trend (due to the effect of ITO layers) around the voltage of 1 V. As a result, the equal absorption coefficients of the two arms can be guaranteed while a large change of $\Re (n_{\mathrm{eff}})$ is attained. In this regard, the color plots of $\Re (n_{\mathrm{eff}})$ and $\Im (n_{\mathrm{eff}})$ of the proposed waveguide as functions of $N_0$ and the external voltage (${{\mathrm {V}}}_s$) are calculated and shown in Fig. 3a and b, respectively. In order to make the variations intelligible, the real and imaginary parts of $n_{{{\mathrm {eff}}}}$ at lower and upper bounds of $N_0$ (i.e. $N_0=2\times 10^{20} \, {{\mathrm {cm}}}^{-3}$ and $5\times 10^{20} \, {{\mathrm {cm}}}^{-3}$) are extracted from Fig. 3a and b which are plotted in Fig. 3c. The most notable point about Fig. 3c is that as we expected, the imaginary part of $n_{{{\mathrm {eff}}}}$ starts to roll up at a specific voltage when the initial doping of ITO increases. Therefore, two voltages with equal $\Im (n_{\mathrm{eff}})$ for the off state of the modulator can be found but this condition occurs for various values of $N_0$.

**Figure 4:**
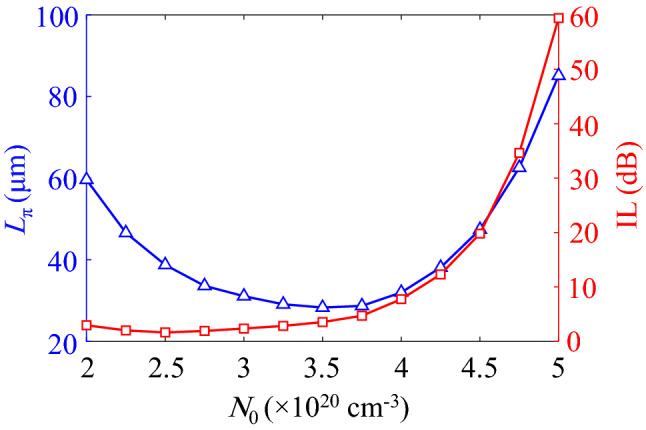
Required arm length for $\pi$ phase shift and its associated IL as functions of ITO initial doping.

In order to find the optimum $N_0$ and off-state voltages, the arm length of the modulator and its associated IL are calculated for various $N_0$ that are shown in Fig. 4. As it can be seen, when the ITO carrier concentration grows, the IL of the waveguide increases because the ITO layers tend to exhibit their metallic characteristics. On the other hand, the shortest required arm length of the MZM could be achieved at $N_0=3.25\times 10^{20} \, {{\mathrm {cm}}}^{-3}$ where the corresponding IL is 2.76 dB.

**Figure 5:**
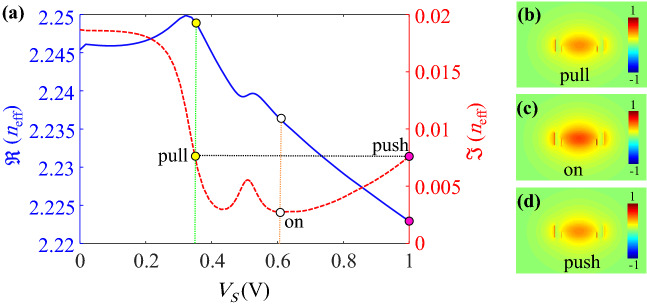
(**a**) $\Re (n_{\mathrm{eff}})$ and $\Im (n_{\mathrm{eff}})$ of the proposed waveguide at $N_0=3.25\times 10^{20} \, {{\mathrm {cm}}}^{-3}$ and as functions of the applied voltage. The guided mode electric field profiles in (**b**) pull, (**c**) on and (**d**) push states on the color scales of $-1$ to 1 V/m.

The characteristics of the guided mode at the optimum initial doping and as a function of the applied voltage is plotted in Fig. 5a. As it is shown, the on-state external voltage should be set at $V_{{{\mathrm {on}}}}=0.607$ V where the guided mode effective index is $2.2377+j0.00264$. Additionally, the applied voltages to the upper (push) and lower (pull) arms are respectively taken as $V_{{{\mathrm {push}}}}=1$ V and $V_{{{\mathrm {pull}}}}=0.366$ V. The corresponding effective indices of the upper and lower arms with equal imaginary parts are $2.249+j0.00763$ and $2.223+j0.00763$, respectively. The guided mode electric field profiles of the waveguide in the pull, on and push states are also depicted in Fig. 5b–d, respectively. Now, the optimization of the MZM should be investigated.

### Waveguide optimization and modulation performance

**Figure 6:**
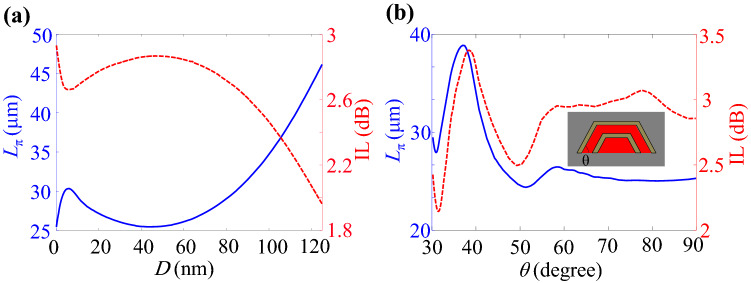
(**a**) Required arm length for $\pi$ phase shift ($L_{\pi }$) and its associated IL as functions of aSi thickness. (**b**) $L_{\pi }$ and IL versus the side wall angle. The inset shows the cross section of waveguide where the Si, stack of thin materials and $\hbox {SiO}_2$ are respectively represented by red, brown and grey colors.

One of the geometrical parameters that can affect the modulator characteristics is the thickness of aSi (*D* in Fig. 1b). Therefore, the required length and IL of the modulator as functions of *D* are calculated and shown in Fig. 6a. As it is shown, when the thickness of aSi decreases from 120 nm, the arm length is reduced down to $25.2 \, \upmu \hbox {m}$. This is due to the fact that the lower *D* leads to more confinement of guided mode and as a result higher light-matter interaction so that a larger phase shift is acquired. On the other side, as it is expected, the IL of the modulator increases from 2 dB at $D=120 \, \hbox {nm}$ to 2.85 dB at $D=43.3 \, \hbox {nm}$.

Another phenomenon that should be noticed is the electrostatic effect of the upper graphene sheet on the carrier concentration of the lower ITO layer as a consequence of thin aSi. As it is shown in Fig. 6a, at the thicknesses lower than $D\approx 4-7$ nm, there exists growth of IL which is attributed to the additional carriers in the ITO layer induced by the upper graphene sheet.

The important point that should be taken into account is that it is not practically possible to place the stack of layers on the Si ridge with vertical side walls. Therefore, a trapezius with the side wall angle of $\theta$ are plotted in Fig. 6b. As it is seen, the parameters do not change significantly for the angles larger than about 60 degrees which assures us that our proposed waveguide design is feasible.

**Figure 7:**
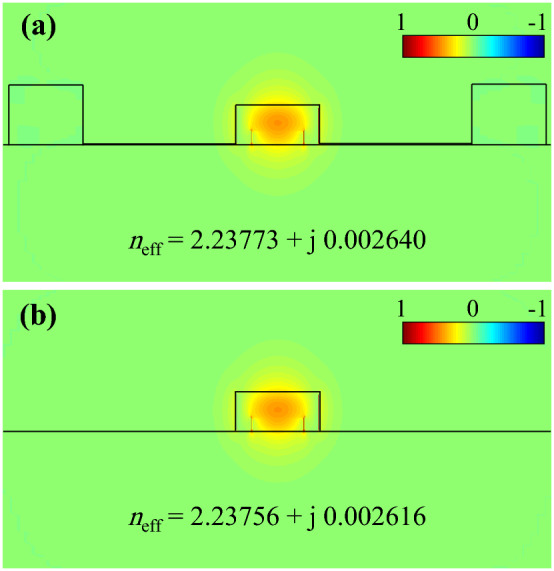
The calculated electric field profiles and effective indices of guided modes for the waveguide (**a**) with and (**b**) without the presence of electrodes.

It is instructive to investigate the effect of electrodes on the device operation. The metal electrodes are located at the two sides of the waveguide to apply the required external voltages. Such metal electrodes can possibly increase the IL of the device. The electrodes are assumed to be 1200 nm away from each other in our simulations. The guided modes of the proposed waveguide in the presence of the electrodes and without electrodes are calculated and respectively shown in Fig. 7a and b. The corresponding guided mode effective indices are also indicated within the figures. As shown in Fig. 7, the imaginary part of the effective index, which is directly related to the IL, is not considerably affected by the electrodes and the change is very negligible because the electrodes are far away from the waveguide.

**Figure 8:**
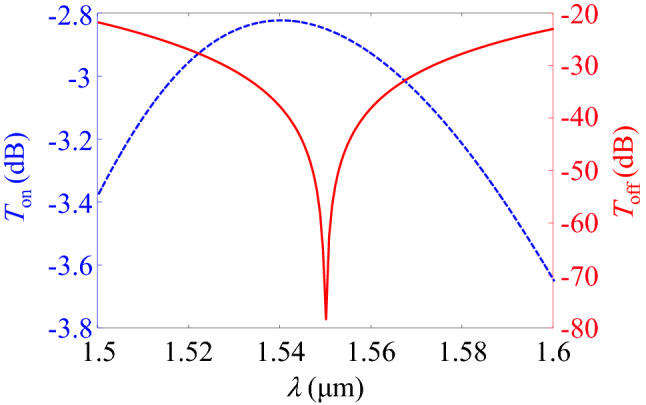
Transmission spectra of the proposed MZM in the on and off states.

Now, the operation of the modulator with the designed waveguide is investigated. The transmission spectra of the MZM in the on and off states are shown in Fig. 8. As it is shown, the IL of the modulator at the wavelength of $1.55 \, \upmu \hbox {m}$ is 2.8 dB. Moreover, the extinction ratio (ER) of the MZM at $\lambda =1.55 \, \upmu \hbox {m}$ is calculated to be more than 30 dB. This is the highest ER ever reported in the literature for a MZM which confirms the outstanding performance of our proposed modulator. It is also interpreting that the ER of the modulator within the whole optical C band is larger than 25 dB that is accompanied by IL lower than 3 dB which guarantees the operation over a wide spectral width.

**Figure 9:**
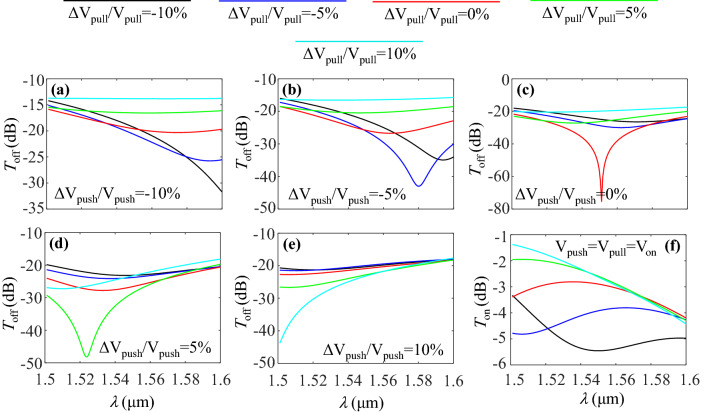
(**a**) Variations of the transmission spectra of the modulator in the off state for various pull voltage errors and at push voltage errors of (**a**) − 10%, (**b**) − 5%, (**c**) 0, (**d**) 5% and (**e**) 10%. (**f**) Transmission spectra of the modulator in the on state for various voltage errors.

For the sake of completeness, the effect of applied voltage errors on the performance of the modulator is studied. In this regard, the transmission spectra of the modulator in the off state for various pull voltage errors at $\Delta V_{{{\mathrm {push}}}}/V_{{{\mathrm {push}}}}=-10\%$, $-5\%$, 0, 5% and 10% are calculated and plotted in Fig. 9a–e, respectively. According to the figures, it is obvious that there are red shifts and blue shifts of the transmission valley by the variations of the voltages. This is due to the variations of $\Re (n_{\mathrm{eff}})$ in the MZM arms so that the required $\pi$ phase shift occurs at different wavelengths. However, in the worst case of $\Delta V_{{{\mathrm {push}}}}/V_{{{\mathrm {push}}}}= \Delta V_{{{\mathrm {pull}}}}/V_{{{\mathrm {pull}}}}=-10$%, the off-state transmission is less than $-12$ dB which can still lead to a high ER. The effect of voltage errors on the on-state transmission of the MZM is also shown in Fig. 9f. As it can be seen, the more the voltage errors move toward negative values, the IL of the modulator increases.

Finally, other electrical characteristics of the proposed modulator are calculated. One of the most important parameters is the bandwidth of the modulator which is limited by the RC time constant of the modulator. The total capacitance of the proposed waveguide is the parallel combination of the two oxide capacitances ($C_{{{\mathrm {ox}}}}$). However, it should be noted that there is also a quantum capacitance ($C_Q$) associated to each of the graphene sheets which is in series connection with each of the oxide capacitances. According to Eq. (4) (see “Methods”), the quantum capacitance per unit area in a single graphene sheet is obtained in the order of $15.8 \, \upmu \hbox {F}/\hbox {cm}^2$. On the other hand, each of the oxide capacitances per unit area is calculated to be $4.425 \, \upmu \hbox {F}/\hbox {cm}^2$. Consequently, assuming that the electrodes are 1200 nm away from each other, the total capacitance of the structure is approximately $44.2 \, \hbox {fF}/\hbox {cm}^2$.

The total resistance of the waveguide is also obtained by adding the graphene sheet resistance to the graphene- and ITO-contact resistances. Assuming a sheet resistance of $100 \, \Omega /\square$^54, 55^, and a graphene-contact resistance of $200 \, \Omega \upmu \hbox {m}$^55, 56^, the total ohmic resistance is obtained as $\sim 430 \, \Omega$ which results in a 3 dB bandwidth of 11.3 GHz. Furthermore, according to the required voltages for each arm, the total energy consumption of the modulator is estimated to be as low as 10 fJ/bit. It should also be emphasized that the required voltage changes of ${{\mathrm {V}}}_{\pi }$ and arm length of $L_{\pi }$ results in a very low ${{\mathrm {V}}}_{\pi }L_{\pi }$ of $10 \, \hbox {V} \upmu \hbox {m}$. According to Table 1 and to the best of our knowledge, this is the lowest ${{\mathrm {V}}}_{\pi }L_{\pi }$ ever reported for the state-of-the-art MZM which reveals the superior performance of our work.

**Table 1:** Comparison of characteristics in related works.

Reference	$V_{\pi } L_{\pi }$ ($\hbox {V} \upmu \hbox {m}$)	$L_{\pi } \, (\upmu \hbox {m})$	IL (dB)	ER (dB)	$\lambda _{opr}$ (nm)	BW (GHz)	Configuration	Sim./exp.
Shu et al.^57^	1290	40	–	25	1550	–	Mach–Zehnder	Exp.
Yang et al.^58^	43.5	43.5	1.37	34.7	1550	–	Mach–Zehnder	Sim.
Wolf et al.^59^	1000	1100	8	14	1550	25	Mach–Zehnder	Exp.
Amin et al.^60^	95	$<3$	9	2	1550	$\sim 1.1$	Plasmonic Mach–Zehnder	Exp.
Current work	10	25.2	2.8	$> 30$	1550	11.3	Mach–Zehnder	Sim.

## Methods

The simulation results in “Results” are based on the finite difference eigenmode (FDE). In this regard, the complex dielectric constant of graphene is obtained from^61^:

1 $$\begin{aligned} \varepsilon _{g} (\omega )=1+ \frac{i\sigma (\omega )}{\omega \varepsilon _0 \Delta } \end{aligned}$$

and imported to the simulations as the optical model. The parameter $\Delta$ is the effective thickness of graphene that is taken as 0.34 nm, $\varepsilon _0$ is the permittivity of free space, $\omega$ is the angular frequency and $\sigma$ is the complex conductivity that is calculated by the Kubo formula^61, 62^

2 $$\begin{aligned} \sigma (\omega )=\frac{j e^{2} \mu }{\pi \hbar ^{2}\left( \omega +j \tau ^{-1}\right) }+\frac{j e^{2}}{4 \pi \hbar } \ln \left( \frac{2\left| \mu \right| -\hbar \left( \omega +j \tau ^{-1}\right) }{2\left| \mu \right| +\hbar \left( \omega +j \tau ^{-1}\right) }\right) \end{aligned}$$

where the first and second terms on the right hand side are respectively the intraband and interband conductivities, $\hbar$ is the reduced plank constant, *e* is the electron charge, $\tau$ is the relaxation time that is assumed to be 0.5 ps^61^ and $\mu$ is the chemical potential. As a result and according to $\tau =\frac{\mu \mu _c}{ev^2_F}$, the average value of graphene mobility in the ON state is approximately $12000 \, \hbox {cm}^2$/v.s.

The dielectric constant of ITO is calculated from the Drude model where the ITO carrier concentration is required. The induced carrier concentration in ITO is calculated according to:

3 $$\begin{aligned} N_{{{\mathrm {acc}}}}=N_0+\frac{C_{{{\mathrm {ox}}}} C_Q }{C_{{{\mathrm {ox}}}} + C_Q } \frac{V_s}{t_{{{\mathrm {acc}}}}} \end{aligned}$$

where $N_{{{\mathrm {acc}}}}$ is the carrier concentration in the accumulation layer with the assumed thickness of $t_{{{\mathrm {acc}}}}=1 \, \hbox {nm}$^63^. Here, $N_{0}$ is the initial carrier concentration of ITO that is assumed to be $10^{20} \, \hbox {cm}^{-3}$, $V_s$ is the external voltage, $C_{{{\mathrm {ox}}}}$ is the oxide capacitance of the ITO-graphene capacitor and $C_Q$ is the quantum capacitance of graphene that is obtained from^43^:

4 $$\begin{aligned} C_{Q}=\frac{2 e^{2} k_{B} T}{\pi \left( \hbar v_{F}\right) ^{2}} \ln \left[ 2\left( 1+\cosh \frac{\mu }{k_{B} T}\right) \right] \end{aligned}$$

where $k_B$ is the Boltzmann constant. The corresponding chemical potential of graphene at a specified $V_s$ is calculated by:

5 $$\begin{aligned} \mu = \hbar v_F \sqrt{\pi \frac{\varepsilon _0 \varepsilon _r}{d_{{{\mathrm {HfO_2}}}}}} \left( V_s-\frac{\mu }{e}\right) . \end{aligned}$$

Then, the calculated chemical potential is applied to Eq. (4) and finally the ITO carrier concentration and dielectric constant are obtained for a given quantum capacitance. The corresponding refractive indices of silicon, hBN, $\hbox {HfO}_2$, Au and Ti are respectively taken as 3.45, 1.91, 1.88, $0.524+i10.742$, $3.684+i4.608$.

Regarding the analysis of the MZM, the transfer matrices of input Y-junction, the arms and output Y-junction are respectively expressed as:

6 $$\begin{aligned} T_{\mathrm{{in}}}= & {} \left[ \begin{array}{c c} \frac{1}{\sqrt{2}} \\ \frac{1}{\sqrt{2}} \end{array} \right] \end{aligned}$$

7 $$\begin{aligned} T_{\mathrm{{arm}}}= & {} \left[ \begin{array}{c c} e^{-j\phi _u} \, e^{-\alpha _u L_{\pi }/2} &{} 0 \\ 0 &{} e^{-j\phi _d} \, e^{-\alpha _d L_{\pi }/2} \end{array} \right] \end{aligned}$$

8 $$\begin{aligned} T_{\mathrm{{out}}}= & {} \left[ \begin{array}{c c} \frac{1}{\sqrt{2}}&\frac{1}{\sqrt{2}} \end{array} \right] \end{aligned}$$

where $\phi _u$ and $\phi _d$ are the acquired phase shifts within the up and down arms. The $\alpha _u$ and $\alpha _d$ are the corresponding absorption coefficients of the arms and $L_{\pi }$ is the arm length.

The output power of the MZM can be obtained by:

9 $$\begin{aligned} \begin{aligned} E_{\mathrm{{out}}}= \left[ \begin{array}{c c} \frac{1}{\sqrt{2}}&\frac{1}{\sqrt{2}} \end{array} \right] \left[ \begin{array}{c c} e^{-j\phi _u} \, e^{-\alpha _u L_{\pi }/2} &{} 0 \\ 0 &{} e^{-j\phi _d} \, e^{-\alpha _d L_{\pi }/2} \end{array} \right] \left[ \begin{array}{c c} \frac{1}{\sqrt{2}} \\ \frac{1}{\sqrt{2}} \end{array} \right] \end{aligned} \end{aligned}$$

which leads to:

10 $$\begin{aligned} \begin{aligned} T=\left| \frac{E_{{{\mathrm {out}}}}}{ E_{{{\mathrm {in}}}}} \right| ^2=\frac{1}{4} \left( e^{-\alpha _u L_{\pi }} + e^{-\alpha _d L_{\pi }} + 2e^{-(\alpha _u+\alpha _d) L_{\pi }/2} \cos (\Delta \phi )\right) \end{aligned} \end{aligned}$$

where $\Delta \phi$is the phase difference between the propagating lights inside the two arms.

## Conclusion

In summary, we proposed a very high-performance electro-optic ITO/graphene-based MZM. According to the presented analysis, the contributions of the plasma dispersion effects in graphene and ITO layers are engineered so that a large difference between $\Re (n_{\mathrm{eff}})$ of the push and pull arms is obtained. Meanwhile, the values of $\Im (n_{\mathrm{eff}})$ in the two arms are equal in the off state. The optimization of waveguide leads to the lowest value of ${{\mathrm {V}}}_{\pi }L_{\pi }=10 \, \upmu$m ever reported in the literature. The simulation of MZM reveals a very high ER of 25 dB and low IL below 3 dB over a wide spectral width that covers the whole optical C-band. Furthermore, the 3 dB bandwidth of the modulator is calculated to be as high as 6.5 GHz and a low energy consumption of 10 fJ/bit is also estimated. The outstanding characteristics of the proposed MZM make it a suitable candidate for integrated optical systems.

## Data Availability

The datasets and materials used and/or analyzed during the current study are available from the corresponding author on reasonable request.
